# Reasons for nonparticipation in the Valuing Active Life in Dementia randomised controlled trial of a dyadic occupational therapy intervention: An interview study

**DOI:** 10.1177/2050312120958926

**Published:** 2020-10-15

**Authors:** Jacqueline Mundy, Jacki Stansfeld, Martin Orrell, Martin Cartwright, Jennifer Wenborn

**Affiliations:** 1 Essex Stroke Hub Team, North East London NHS Foundation Trust (NELFT), London, UK; 2 School of Health Sciences, City University of London, London, UK; 3 Research and Development Department, North East London NHS Foundation Trust (NELFT), London, UK; 4 Institute for Mental Health, University of Nottingham, Nottingham, UK; 5 Division of Psychiatry, University College London, London UK

**Keywords:** Decliner, dyadic, dementia, qualitative interview, thematic analysis

## Abstract

**Objectives:**

There is currently little known about why people decline to participate in dyadic, psychosocial dementia research. This interview study aims to explore the reasons why people declined to participate in the Valuing Active Life in Dementia research trial.

**Methods:**

Ten family carers of people with dementia, who were part of a dyad that had declined to take part in the randomised controlled trial, participated in qualitative telephone interviews to explore their reasons for declining. Inductive thematic analysis was used to identify themes.

**Findings:**

Two themes with related sub-themes were identified: (1) Protectiveness – protecting the person with dementia, themselves as carers and their current lifestyle; (2) ‘It’s not for us’ – the time commitment, and the possible unsuitability of the intervention, was seen to outweigh the perceived benefit of taking part. People with dementia were not always involved in the decision-making process, with carers stating the decision not to participate was made in the usual way as all their decisions. No apparent differences between the spousal and the child carers were apparent in the small sample.

**Conclusion:**

Recruitment to randomised controlled trials can be considered difficult or unfair because some participants will miss out on the desired intervention. However, this study shows that concern about the time and inconvenience of being involved in the trial can put people off research participation. Identifying possible reasons for declining research participation contributes to the design of future trials and recruitment strategies, so that the potential benefit is considered relative to the time and effort involved. Offering research opportunities to people with dementia and their families at the right stage of the dementia trajectory for their needs, facilitating personalised recruitment strategies with finely tailored researcher communication skills should help maximise recruitment, reduce attrition and deliver a more successful trial.

## Background

With increasing numbers of people living with dementia as the population ages, there is an urgent need to improve dementia care and support in the community through research investment.^1^ This investment therefore needs participation from people living with dementia and their family carers.

Dementia symptoms can include memory loss, mood changes and communication difficulties bringing daily challenges and increasing reliance on family for support.^2^ Adjusting to their loved one’s declining cognition and functional abilities can be both demanding and distressing. Many interventional dementia studies are dyadic due to the nature of the dementia symptoms.^3^ Moreover, carers’ support can be critical in facilitating the person with dementia’s decision-making and participation in research and interventions.^4,5^ However, the motivation to participate in dyadic psychosocial dementia research is largely uninvestigated.^6^ Different types of dyadic relationships may affect the willingness to engage in dementia research.^4,7^ Elad et al.^6^ suggest the higher proportions of spousal research participants may reflect a higher commitment to the person with dementia from their spouse and/or greater time limitations in adult children. Wimo et al.^8^ identified two-thirds of participants in a European survey were spousal with most being wives. However, whether participation in research is representative of societal carer relationships in psychosocial research is not known.

The Consolidated Standards of Reporting Trials (CONSORT) require the proportion of people who declined to participate to be reported.^9^ This reporting allows trial rigour to be assessed by indicating whether participants represent societal prevalence or if sections of the cohort are unaccounted for. However, the numbers of decliners alone does not reveal their decisions for nonparticipation. Reasons can be multifaceted, incorporating the perceived acceptability of the research processes, the intervention, personal risks and implications together with lifestyle considerations.^10^ The different levels of commitment, risk and perceived benefit for both parties suggest that motivators for and against participation differ. It may involve differing opinions and conflicting obligations within the dyad, thus making decisions more complex than nondyadic research participation.

The ethical principles underpinning the right to refuse research participation are established in the Declaration of Helsinki.^11^ Furthermore, people are not obliged to explain their reasons for declining.^12^ These principles can restrict the reporting of why people decline.^13^ Some reasons for declining psychosocial dementia trials are reported, either with the trial outcome^5,14–19^ or when reflecting on recruitment issues.^20,21^ These papers reported multifaceted reasons for declining, including time constraints, the wish to maintain normality, perceived insufficient value exchange, dissonance between dyad’s health and the study requirements, and protection of the person with dementia. Reports also state that many people do not provide a reason for declining, although it is unclear how formally the information was obtained. Qualitative exploration of why people decide not to participate in dyadic psychosocial research has not been undertaken with semi-structured interviews, but has the potential to identify barriers to participation. Reporting these barriers can improve the acceptability and inclusivity of future study designs for people with dementia and their family carers.

The main aim of this study was to identify and explore, from the family carer perspective, why dyads decided not to participate in the Valuing Active Life in Dementia (VALID) randomised controlled trial (RCT) (ISRCTN 10748953).^22^ The secondary aim was to identify if the reasons given differed according to the carer’s gender or type of relationship with the person with dementia, that is, spousal or nonspousal.

## Methodology

### VALID randomised controlled trial

This qualitative study was embedded within the multi-site, pragmatic, two-arm, parallel group, single-blind individually randomised VALID RCT. The RCT aimed to establish the clinical and cost effectiveness of the Community Occupational Therapy in Dementia – UK version (COTiD-UK) intervention compared to treatment as usual. Dyads (pairs) comprising a person with dementia and a family carer were recruited, via memory services and other relevant health and social care services that support people with dementia living in the community and their families. The former had to live in their own home; have a diagnosis of dementia as defined by the *Diagnostic and Statistical Manual of Mental Disorders* (4th ed.; DSM-IV);^23^ and score between 0.5 and 2 on the Clinical Dementia Rating Scale indicating mild-to-moderate dementia.^24^ Carers had to be aged 18 or above and provide practical support with domestic or personal activities to the person with dementia for at least 4 h per week. Both parties had to be able to converse in English; be willing to participate in the COTiD-UK intervention together if allocated to receive it; and have the capacity to provide consent. Face-to-face research assessments took place with the dyad in the person with dementia’s home pre-randomisation, at 12 weeks and 26 weeks post-randomisation, and then by telephone with just the carer at 52 and 78 weeks post-randomisation. Dyads were randomly allocated to either receive the COTiD-UK intervention or the usual service provided within their locality, which may or may not include occupational therapy.

The aim of COTiD-UK is to maximise the ability of the person with dementia to engage in personally meaningful activities and improve the carer’s sense of competence. The 10-week intervention is delivered to the dyad together in the person with dementia’s home. It incorporates individual narrative interviews, setting personalised goals reflecting the dyad’s interests and aspirations and working with the occupational therapist to achieve them. Goals can include the development of coping strategies, environmental adaptation and the maintenance of hobbies and activities.

A total of 15 research sites were recruited across England between September 2014 and May 2017, although one site did not proceed to recruiting pairs due to a major service reorganization; 468 dyads were recruited from the remaining 14 sites between September 2014 and July 2017.

### Design

This was a qualitative study using semi-structured telephone interviews with a convenience sample of family carers of people with mild-to-moderate dementia who had declined to take part in the VALID RCT and used inductive thematic analysis to identify themes from the data collected.^25^

### Participants

Family carers who had expressed interest in the VALID RCT and been provisionally assessed by the local researcher as being eligible to take part, but then declined to do so, were eligible to take part in this qualitative interview study. Carers who had been screened as not being eligible for the VALID RCT were not eligible for this qualitative study.

### Recruitment

Four of the VALID RCT sites took part in this qualitative study. The sites were selected because they were recruiting to the RCT during this period and had the local researcher capacity and willingness to manage the additional workload, and to maximise the geographical spread of the sample. Local site researchers were asked to identify potentially suitable participants while screening and recruiting participants to the VALID RCT. The RCT screening usually took place via a telephone call to the carer following their expression of interest in taking part and having been provided with the RCT recruitment materials.

The recruitment process for this qualitative study was outlined in the VALID RCT Trial Operations Manual. A document, approved by the Ethics Committee, was provided to enable local researchers to adopt a consistent method of approaching potential participants, and to gain verbal consent for the use of some screening data to be retained, namely the carer’s gender, relationship to the person with dementia and their postcode. The local researcher asked the following question at the point at which a family carer declined to participate in the RCT:

We would like to understand why family carers decide not to take part in VALID, this is so we can make research more accessible to people with dementia and their family carers in the future. Do you mind telling me why you decided not to take part?

The researcher categorised the response using a predefined list of possible reasons that could be read out to the carer to choose from if they wished. Reasons for declining were as follows: ‘We are managing fine at the moment’, ‘We/I do not wish to take part in research in general’, ‘We/I am too busy’, ‘We/I am not well enough for VALID’, ‘We/I don’t like the idea of a 50:50 chance of receiving the occupational therapy or not’, ‘Other–please give details’ and ‘I do not want to give a reason’. Analysis of these data was planned to enable interpretation of the reasons for declining.

If the family carer agreed to have the above information recorded, the researcher then continued by saying,

Thank you. We would be very interested to hear more about your reason/s for not wanting to take part in VALID; this would involve a short telephone conversation with Jacqueline Mundy, a nurse researcher at a time convenient to you. Can I pass your details to her so she can contact you to arrange this?

If the carer declined at this point, they were thanked for their time and no further action was taken. If the carer did agree, their name, postal address and telephone contact details were recorded and the completed form sent via a secure email portal to the first author.

This process was standardised but also dependent on the local researcher’s assessment and judgement of the individual situation, for instance, the carer’s current level of stress and competing demands on time. So if at any point in the process, the local researcher, who was trained and experienced in recruitment processes and often from a clinical background, felt that the questions were intrusive or ill-timed for that individual at that time, then the above process was not followed. Examples of situations when this process did not happen included the following: the contact was with the person with dementia who then declined involvement and it was not possible to speak to the carer, the carer expressed feelings of stress or reported feeling unwell and it was therefore unethical to add to their burden, or having declined participation in the RCT the carer terminated the call before the researcher could proceed. No further contact was made following the point at which the dyad reported their decision to decline the RCT as that was considered intrusive and unethical bearing in mind they had declined research involvement at that point.

Between January and September 2016 at the four participating sites, 146 dyads who had been initially screened as being eligible to take part in the RCT declined to do so. However, it is not known how many of these would subsequently have been eligible for the RCT; were actually asked to share their reason(s) for declining; did or did not do so; or were then invited to take part in the telephone interview, as local researchers varied in adhering to the process outlined.

On receipt of the completed form, Jacqueline Mundy then made contact by telephone, introduced herself as the VALID Trial Manager and explained the purpose of the interview. If the carer agreed to be interviewed, a consent form with a prepaid return envelope was posted, for completion and return prior to the telephone interview.

Jacqueline Mundy received 18 expressions of interest via local researchers to make contact with. One person could not be contacted; two then declined to be interviewed with no reason stated; and three declined to be interviewed stating they were too busy. Twelve carers agreed to participate and returned a consent form. One then declined to participate as they had experienced a dementia crisis at the time of being screened for the RCT and decided they did not want to revisit that difficult time. The second was then in a crisis situation at the time arranged for the interview and so was unable to proceed.

### Data collection

#### Interview Topic Guide development

Jacqueline Mundy, Martin Orrell and Jennifer Wenborn developed an indicative Topic Guide for the semi-structured interviews. This guide comprised a series of key questions along with suggested prompts, to explore dyads’ reasons for declining to take part in the VALID RCT and the decision-making processes they had used. The questions were based on Connell et al.’s^13^ focus group study of American family caregivers’ attitudes towards people with Alzheimer’s disease taking part in clinical research, and then refined through the authors’ extensive experience of screening and recruiting potential participants for the VALID RCT and other dementia intervention studies.^22,26,27^ The study participant recruitment documents and interview Topic Guide were reviewed by the VALID Public and Patients Involvement (PPI) reference group. This group comprised three former spousal carers of people living with dementia, some of whom went on to also have caring roles for other family members. The PPI group was involved throughout the 6-year VALID research programme and therefore had a good knowledge of the research aims, activities and the COTiD-UK intervention. The recruitment materials and the Topic Guide were amended in response to their comments and suggestions to enhance the relevance and clarity.

#### Topic Guide content

The Topic Guide included the following questions: (1) What were your views about taking part in research before being invited to VALID?; (2) What did you think taking part in the VALID study would involve for you both?; (3) How did you come to the decision not to take part in VALID?; (4) The reason you gave for not taking part was (reason as recorded by the local researcher inserted here), please could you tell me more about this?; (5) What influence did the initial approach have on your decision not to take part?; and (6) What would make taking part in research easier for your particular circumstances? Additional prompts were included in the Topic Guide to facilitate obtaining the dyad’s views and beliefs relating to research, as stated by the carer. The use of active listening, probing, and open and closed questions enabled further exploration of participants’ views as required. Interviews were intentionally designed to be short in order to reduce impact and inconvenience, bearing in mind the participants had previously declined to take part in a research study.

#### Interviewer

Jacqueline Mundy conducted all the interviews. She is a registered general nurse with extensive clinical experience and research experience of working with people with a diagnosis of dementia and their family carers. Jacqueline Mundy was the VALID RCT Trial Manager at the time of conducting the interviews but had no previous contact with the interviewees before making contact with them regarding this qualitative study. She completed the study in part-fulfilment for a master’s degree in Health Science Research.

#### Interviews

Jacqueline Mundy conducted the telephone interviews within 2 weeks of the interview consent form being returned, at a time convenient for the interviewee. The interviews lasted between 6 and 38 min, with the duration dependent on the interviewee’s availability and contribution. Interviews were audio-recorded using an encrypted recorder and stored securely in password protected files. Field notes were also made immediately after each interview.

### Data analysis

The audio-recordings were transcribed verbatim, checked for accuracy and anonymised by Jacqueline Mundy. Inductive thematic analysis was selected in order to generate themes from the data rather than being shaped by prior assumptions or theories.^25^

The initial analysis was performed by the first author and began with data familiarisation through reading and re-reading the transcripts and noting items of potential interest. Relevant patterns of meaning, including words and brief phrases, were identified and an initial coding framework was developed. To maximise the rigour of the coding process, a second researcher (Jacki Stansfeld) contributed to the data analysis. Jacqueline Mundy and Jacki Stansfeld agreed a coding framework and then independently coded all the transcripts, meeting as necessary to review and refine the codes, and discuss discrepancies until agreement was reached. Jacki Stansfeld was unaware of the aim of the analysis to maximise inductive coding and reduce bias. This iterative process continued, with themes being identified and then refined through discussion between Jacqueline Mundy, Jacki Stansfeld, and Jennifer Wenborn until agreement was reached. Data from wives, husbands and adult children were analysed separately to identify any differences that were potentially attributable to the carer’s gender or relationship with the person with dementia. All quotations have been anonymised, providing nonspecific demographic information to maintain confidentiality.

Ethical approval for the VALID RCT, which included this embedded qualitative study, was granted by the London – Camberwell St Giles Research Ethics Committee (Reference 14/LO/0736 on 14 July 2014). The additional participant recruitment materials, consent form and interview Topic Guide were subsequently approved as a Substantial Amendment on 10 December 2015.

All participants provided written informed consent before taking part. All data were stored in line with NHS Data Protection requirements, and the study was conducted according to Good Clinical Practice (GCP) and the study sponsor standard operating procedures.^12^

## Findings

A total of 10 family carers participated in the telephone interviews. The majority were female (7/10) and had spousal relationships (7/10) with the person with dementia; three were adult child carers (one daughter, one niece and one interviewee who cared for both her father and mother-in-law). No other participant characteristics were recorded. Table 1 summarises the participants’ characteristics.

**Table 1.** Participant characteristics: ID, gender and relationship to the person with dementia.

ID	Gender	Relationship to person with dementia
01	Male	Husband
02	Female	Daughter
03	Female	Wife
04	Female	Niece
05	Male	Husband
06	Male	Husband
07	Female	Daughter/daughter-in-law
08	Female	Wife
09	Female	Wife
10	Female	Wife

All interviewees expressed interest in taking part in dementia research in general. Six carers reported that a lack of interest in research by the person with dementia was the reason for declining to take part in the VALID RCT, and eight reported having current and/or past experience of taking part in research.

All participants were asked: ‘how did you come to the decision not to take part in VALID?’ Carers responded that the decision not to participate was made in the same way that the dyad usually made decisions, that is, if the person with dementia usually led the decision that continued. Decisions were not always made jointly; most were carer led (n = 7), two were led by the person with dementia and one carer was unable to discuss participation as the person with dementia immediately dismissed the idea. Three instances of joint discussion were reported with two led by the person with dementia and one by the carer, and three carers had discussed participation with another family member before discussing it with the person with dementia. Three carers had decided alone, of whom one spoke to the person with dementia afterwards.

Three carers offered suggestions in response to the final question asked: ‘What would make taking part in research easier for your particular circumstances?’. All the suggestions were linked to their stated reason for declining: first, the need to continually raise the awareness of dementia so as to reduce stigma; second, for research to be more flexible and require less time from the participants so it was less impactful on an already busy lifestyle; and third, to provide a more incremental introduction to research so as to minimise the risk of the person with dementia being overwhelmed with too much information.

## Themes

Two main themes were identified in relation to whether taking part in the study was considered ‘worth it’: (1) Protectiveness and (2) ‘It’s not for us’. These reflected balancing the need to protect the person with dementia, themselves and their current lifestyle, that is, maintaining the status quo, versus the time and disruption of taking part and how suitable the intervention was perceived to be for their particular circumstances. These conflicting themes resulted in a perceived imbalance of benefit in return for their involvement in the trial. Table 2 summarises the themes and sub-themes that are then discussed below.

**Table 2.** Themes and sub-themes.

Theme	Sub-themes
Protectiveness	Protecting the person with dementia
	Protecting themselves as carers
	Protecting the dyad’s way of life
‘It’s not for us’	Time and disruption
	Intervention

### Protectiveness

Protecting the person with dementia, themselves and the dyad’s current lifestyle was evident throughout the interviews.

#### Protecting the person with dementia

Carers shared strong views about protecting the person with dementia. This included avoiding doing anything that may cause distress, such as taking part in the intervention itself or the research evaluation visits:

If there’s anything we don’t have to do for her . . . then we try not to do it. (04)

Female carers especially reported shielding the person with dementia from any situation that could precipitate any distressing emotion such as embarrassment, frustration or shame. The VALID RCT requires participants to acknowledge and have insight into the diagnosis of dementia. However, three carers reported strong reluctance of their spouse or relative to acknowledge their diagnosis and symptoms, which had therefore blocked participation:

Nobody ever mentions it [dementia] because you don’t mention things like that in front of [person with dementia]. (03)

My husband isn’t interested in talking about it at the moment. (10)

Carers perceived the researcher visits and questions, and the intervention itself, as being situations that may highlight the person with dementia’s forgetfulness, difficulty concentrating or trouble completing complex tasks, and so decided to avoid them, so as to protect the person with dementia:

He is deeply embarrassed by the diagnosis of dementia. (08)

All the female adult child carers reported reflecting on broader considerations than just the participation needed by themselves and the person with dementia, for example, the potential benefit to others. However, protecting the person with dementia had remained their priority consideration:

I want to be helpful, but have we got the time for it and can she cope with all that? (07)

#### Protecting themselves as carers

Carers who reported that they had not discussed the decision about taking part with the person with dementia suggested they had taken this approach to shield the person with dementia from distress, as described above. However, reducing distress in the person with dementia also contributed to reducing their own burden:

A lot of things I can’t discuss with my husband, because he gets a bit worried by it and it plays on his mind and then I hear about it for a long time. (08)

Carers appeared more willing to discuss the practical difficulties of living with dementia than feelings of embarrassment or social undesirability. References to practical concerns as opposed to referring explicitly to carer burden were more freely given by all interviewees. There were no specific references to carer burden in any of the interviews with husbands as the focus of their responses related to the person with dementia rather than themselves. However, there was an implied burden from female interviewees, where they described feeling the need to compensate for the person with dementia’s deficits. Two wives described caring for the person with dementia as an extension of their spousal role and had accepted the inevitable decline:

. . . because it’s happening slowly you learn to deal with it say a month at a time rather than it just collapsing on you . . . I mean we’ve had a good life and everybody has a cross to bear. (03)

#### Protecting the dyad’s way of life

Carers reported the need to preserve the routine and normality they currently had, that is, maintaining the status quo. Introducing the intervention was seen as potentially upsetting that balance. Seven carers inferred and three explicitly stated that they were managing day-to-day at a level they felt was acceptable to maintain normality, and so they did not feel the need for any additional interventions at the time of the VALID RCT invitation:

Very early days for us, we haven’t got any problems. (10)

While the COTiD-UK intervention is a dyadic intervention, aimed at both the person with dementia and the carer working together with the occupational therapist, it seems that carers primarily focused on meeting the person with dementia’s needs and wants, with their own needs taking second place. Female adult child carers reported that they were managing through collaboration with the person with dementia, themselves and other relevant carers:

We are into a routine . . . we are managing everything now. (04)

Seven of the carers described having a structure and using various coping strategies to enable this, for example:

. . . on a Monday morning, the first thing I do is write up a whiteboard for the week . . . (07)

Half the interviewees stated that the intervention being delivered in the person with dementia’s home felt like an invasion of their privacy:

I don’t mind doing the research . . . I’m not really dead keen on somebody coming in and seeing at our lifestyle and trying to adjust things. (03)

### ‘It’s not for us’

This theme related to weighing up the time and effort involved in taking part, and wondering how suitable the intervention might be to their relative with dementia – compared to how much benefit may be obtained.

#### Time and disruption

The potential inconveniences of the time required and disruption to routine were reported. Avoiding taking on additional activities within already busy day-to-day lives was evident in interviews with all the female interviewees:

I couldn’t afford to . . . spend an awful lot of time, more time, adding to what I already do. (09)

For those who reported an anticipated negative impact of VALID on their time, most considered the duration of the intervention as being sufficient reason to decline, without reference to the research assessment measures which may have involved up to an additional 18 months follow up:

10 weeks is a long time to commit yourself. (01)

#### Intervention

Carers listed various factors concerning the COTiD-UK intervention itself that had contributed to their decision to decline participation. These included the following: the timing of the intervention in terms of severity of dementia, as well as its suitability for those with physical or other health conditions; understanding the content of the intervention based on the examples provided in the recruitment materials; and how it compared with other interventions or their own previous experience of occupational therapy.

Some carers questioned if the intervention would be suitable for the stage of dementia their relative was at. Some felt that the person with dementia was too early in their dementia pathway and therefore did not have functional problems to address, while others implied they were too late in its progression to benefit. Half the interviewees questioned if the intervention would be suitable for the person with dementia bearing in mind their level of physical frailty or other health conditions:

As much as she would like to, she wouldn’t physically be able to, not just the mental side of things but the physical as well. (02)

Interviewees voiced reservations about the content of the intervention itself and wondered if it had enough potential benefit to warrant the time and energy required. One interviewee recalled the Participant Information Sheet gave ‘joining groups’ as a potential activity example and had found this particularly unattractive:

I not terribly interested in getting involved [joining groups] with a lot of people like that. My husband certainly doesn’t want to know. (03)

With the COTiD-UK intervention being very personalised, only broad information and a limited list of examples could be provided in the recruitment materials. This was partly so potential participants did not feel overwhelmed by the amount of information, and partly to reduce the risk of dyads allocated to the treatment as usual arm of the trial ‘self-medicating’ by taking up some of the COTiD-UK intervention suggestions. However, this may have led to dyads being unclear as to what the intervention itself may involve, other than it being an occupational therapy intervention which requires a time commitment of 10 h over 10 weeks, with many participants not having any prior knowledge of occupational therapy per se. Furthermore, dementia symptoms can affect the ability to understand information and make decisions, in which case perhaps it was easiest and least confrontational to simply decline participation:

Not working out exactly what is meant, his immediate answer is ‘no, definitely not’. (07)

Only two dyads had previous experience of occupational therapy: one was currently receiving therapy for memory problems and the other had had a negative experience when addressing physical issues, which may have affected their decision to decline:

The last time . . .. it didn’t work. (06)

While there was a reported desire to avoid additional activities and ‘over-medicalising’ lifestyles by taking part in additional health related activities, there was a hope that research participation may lead to a quick fix. There was an expressed confidence in benefits from medication above psychosocial interventions by all husbands and two wife carers – all of whom had experienced improvements in the person with dementia once they had started medication. One husband referred to the great lengths people may go to for a hope of improvement or the ultimate cure:

If they said eat mice droppings you’d try it, wouldn’t you? You know to try and see if it made a difference. (05)

However, he had also reported that the amount of interactions the intervention involved were too long and the number and type of visits restrictive to their lifestyle, which was always active but had improved since starting the dementia medication:

[She] became more active and enjoyed life a bit more when she had the tablets. (05)

## Discussion

This interview study explored why family carers declined to participate in the VALID RCT. It enabled the family carers perspective to be heard, at the point of declining, relative to their specific circumstances and dyadic relationship. It is a small sample related to participation in one specific intervention study; however, the results can potentially inform future study design, recruitment and communication strategies to better fit the demographics, needs and values of people with dementia and their family carers.

It was evident that interviewees valued the benefit to both themselves and others from participation in research. However, there was no single reason given for declining the VALID RCT and no specific reference to trial randomisation or the dyadic participation being an issue. However, protective caregiving and the perceived onerous requirements of both the research interviews and data collection, and the intervention, led to a dissonance between initially expressing interest and then actually taking part.

All interviewees expressed protective tendencies towards the person with dementia. The avoidance of embarrassment and stigma, and maintaining the person with dementia’s dignity, was also evident, which is commonly highlighted as a barrier to research recruitment.^28^ Furthermore, other health issues contributed to not feeling able to take on anything extra, and perhaps this is to be expected in a predominantly ageing cohort with multiple comorbidities. For those in the early stage of dementia, their decisions suggest they are living in the present and are unaware of, or in denial of the degenerative nature of dementia, without any consideration for future need. Carers reported being wary of approaching the person with dementia because of how they may negatively react to the term dementia or the suggestion that they may need help.

Inconveniences, such as time limitations, outweighed any perceived benefit from participating. Inconveniences have previously been cited as a significant barrier to participation in dementia trials.^5,14–21^ It is accepted that not all expressions of interest in trials will convert into participants due to the time constraints inherent in caring.^29^ However, it is possible that inconveniences were considered easier to talk about as reasons for declining than risk opening the ‘Pandora’s box’ of caring for a person with dementia.

Although COTiD-UK is personalised and can be of a shorter duration, the time requirement was considered as too disruptive by over half of the declining interviewees. Practical obstacles for psychosocial interventions including the impact on routine and day-to-day stability have been previously considered burdensome.^21^ Information regarding the time commitment needed for the research assessments in terms of the number and duration of researcher visits, as well as the intervention if allocated to receive it, was included in the RCT Participant Information Sheets. Furthermore, the median time for an occupational therapy assessment/intervention in usual practice is 2.5 h which is considerably shorter than the proposed 10 h of the COTiD-UK intervention in the VALID trial.^22,30^

Some carers considered their relative’s stage of dementia as being too severe for the person to either take part in the trial or to benefit from the COTiD-UK intervention, while others described the person with dementia as being too high functioning and therefore not being in need of the intervention. The COTiD-UK intervention is designed for people with mild-to-moderate dementia and the initial screening process to assess eligibility for the RCT included researchers completing the Clinical Dementia Rating Scale through carer report in order to determine the severity of dementia.^24^ Thus, the person with dementia had been provisionally assessed as having the necessary degree of impairment required, unless of course the dyad had declined before the screening process was finished. This reflects Murphy et al.’s^31^ finding from participants of an interventional dementia study, about how important the timing of recruitment in response to the person’s need for help and motivation to change is. Furthermore, it highlights the importance of optimal engagement and identification of the population most likely to benefit from the intervention, and proactive and purposeful screening to maximise efficient and timely recruitment.

However, in our experience, it is not unusual for carers to either underestimate or overestimate the ability of the person with dementia and make assumptions about what they can and cannot still do. And we have often witnessed carers’ surprise at what the person with dementia can still do and enjoy. The key aim of the COTiD-UK intervention is to identify and then build on the person’s remaining abilities by adapting the environment and enhancing the dyad’s coping strategies to enable more purposeful engagement in their chosen activities. As the intervention goals focus on the dyad’s chosen activities, it is difficult to portray the breadth of activities that may be involved without providing too much information at the recruitment stage. Hence, the few examples that are provided do not appeal to everyone, for example, the participants who were put off by the suggestion of attending a community group.

The secondary aim was to identify if there were differences according to the carer’s gender or relationship with the person with dementia. However, this was not feasible bearing in mind the small sample size. No themes were identified that related to the different dyadic relationships. However, wives may have been more dismissive of the additional support they were providing because they considered caring as an extension of their spousal role, as previously identified.^32^ Furthermore, adult child carers are more likely to have to squeeze caring duties alongside their own family demands possibly with less time for other considerations, including research participation.^33^ It has also been found that while the caring role is challenging, there are also rewarding and satisfying positive aspects to caring.^34^ These more positive aspects of caring were identified mostly by the female child and male spousal carers and may explain some of their unwillingness to seek solutions that may upset that dyadic balance and status quo. Carers generally led on the decision-making process, as found in similar studies.^6^ The decision was not always joint. Weighing up the pros and cons with consideration for the person with dementia as a higher priority than themselves was noticeable. For wives, where the progression of dementia had affected the roles within their relationship such as they now led decision-making, may have been different from their previous dyadic decision-making. Where the severity of the dementia was not so evident, whether to participate was discussed more jointly if the person with dementia was not in denial. In some cases, the carer was wary of causing upset to the person with dementia so they made the decision themselves or with other family members. Decision-making on behalf of the person with dementia was implied as being more difficult by female adult child carers in the interviews. Hirschman et al.^35^ found wives were more likely to include the person with dementia in decision-making than female adult child carers. However, this was not so evident in the interviews. Gant et al.^36^ found male adult child carers report lower distress over memory and behaviour problems, which may be significant in them not seeking innovative solutions via research participation or because of societal expectations of males. Adult child carers reported more overall collaborative decision-making, possibly because of a reversal in the power dynamics of authority within the dyad and as such required more active discussion than established spousal relationships. Carers who did not discuss the opportunity with the person with dementia readily justified their decision.

Participation in research needs to be considered on the individual merits of each study for each individual. Furthermore, acceptability and decision-making processes are enhanced by the provision of clear information, pitched at the right level for potential participants’ needs and lifestyle.^37^ While all interviewees reported they had received enough information to consider the VALID RCT, it remains unknown whether decisions were fully informed with most interviewees reporting unfavourable aspects associated with the more demanding trial arm. This may mean that the randomisation processes and the complex personalised intervention being researched were not fully understood.

## Limitations

The fact that the Trial Manager conducted the interviews may have introduced bias, restricting participants’ responses to socially acceptable practical reasons within the context of the short interviews. However, for all but one interviewee, the Trial Manager was not part of the local research team which potentially made her seem more ‘neutral’. Indeed interviewees may possibly have felt more comfortable talking to someone who they perceived as having the opportunity and power to address inconveniences or issues reported. Also, the interviewer’s focus on practical aspects may have been inherent in the questioning and reporting style used.

The interview inclusion criteria were purposely inclusive to maximise the number of participants. However, this inclusivity may have meant that not everyone interviewed would have ultimately been eligible for the VALID RCT, had they not already declined. While a more purposive sample with an even spread of carer relationships may have been preferable to the convenience sample recruited, it can obviously be difficult to invite someone who has just declined involvement in one study to immediately take part in another study to talk about their reasons. The initial approach for interview was outlined and a script provided, but was conducted by the local VALID researcher who undertook the RCT screening process. It was therefore dependent on the local researcher using their experience to decide if it was appropriate to invite the family carer to take part in the qualitative study following their refusal of the trial and then having the confidence and time to follow this through. Local researchers reported some carers terminated the call directly after declining or the researchers themselves considered invitations for interviews inappropriate. It is therefore not known how many VALID ‘decliners’ were actually eligible for this study, how many were invited to provide a reason for declining, who did or did not, nor how many were then invited to take part in an interview. It therefore means we are not sure how representative of those dyads who declined this small sample is.

The small sample size is acknowledged, and a larger sample would have been preferred, not least to feel that saturation had been reached. However, this study was conducted towards the end of the RCT, and so once recruitment to the RCT ended – as scheduled and agreed with the funder, and having recruited 468 dyads (98% of the target sample) – then this study also ended.

The Topic Guide was developed by the members of the research team based on their research recruitment experiences and informed by the literature but it was not piloted. However, it was reviewed by PPI colleagues involved in the VALID research programme based on their personal experience of being carers of people with dementia and their knowledge of the VALID research programme and activities. They validated that the themes identified reflected their own experiences.

A further potential bias is that the reasons the dyad declined are reported only by the carer and may therefore be their own interpretation rather than a truly dyadic perspective. Some carers reported conflicting feelings between the person with dementia and themselves about taking part in the VALID RCT. This may have been related to the dementia symptoms as identified by Hirschman et al.^35^ or more specifically the partnered component of the study and intervention. While there was no specific dyadic conflict reported, as suggested by Brodaty and Green,^38^ initial discord within the dyad may have resulted in the willingness of carers to participate in the interviews. It is acknowledged, in other health conditions, that individuals with strong feelings are more likely to provide reasons for declining in which case dyads who agreed between themselves not to participate for reasons they both agreed on may have been less inclined to participate in the interviews, potentially affecting the generalisability of findings.^39^

## Future research and clinical implications

Clinical interventions need to be evidence-based via research. Identifying and reporting the number of participants and decliners, and their reasons for participation/nonparticipation, enables the full spectrum of views about the research and intervention to be appraised in context, which in turn can enable future research designs to better meet the needs of potential participants. While there is some evidence of increasing numbers of people participating in dementia research accommodating dyads’ needs better may serve to continue the trend to reach the national 10% target.^1,40^ It is particularly important, in the absence of a cure, to develop acceptable interventions that can enable people living with dementia to do so better and for longer.

It is recommended that data collection, sharing and reporting need to be improved. It is important to collect data from those who decline to participate as to why and consider if their characteristics vary to those who do consent. Reducing some information sharing barriers pre-consent, within the proviso of maintaining appropriate ethical boundaries and data protection requirements, would enable decliners’ views and reasons to be incorporated in all trial findings, in a more consistent and effective way. For example, in this study, decliners had to consent to the three data items to be shared with the lead research site, which affected how much detail could be provided when reporting the RCT. Locally, healthcare institutions could advise their client group that they are research active and may share anonymised reasons given for declining as part of good practice. A more comprehensive approach could be to incorporate nested qualitative interviews in all RCTs with both decliners and participants, to enable a more balanced view of acceptability for implementation purposes. Finally, reporting the data thus collected will increase the validity of the trial results.

Study recruitment strategies and inclusion/exclusion criteria may benefit from early feasibility testing and being more explicitly targeted. Furthermore, streamlined screening processes to efficiently identify people who are most likely to participate in the intervention being evaluated are recommended. Psychosocial interventions require commitment to be effective, and in this small sample, the COTiD-UK intervention was considered as a barrier to participation by some interviewees. Therefore, focusing recruitment on people with dementia and their carers who already participate in community activities may benefit recruitment strategies, although caution would be advised to avoid biasing the sample. Alternatively, consideration at the design stage as to whether participants are able to choose their preferred arm of the trial may encourage more inclusive participation.

Recruitment communication strategies for complex psychosocial interventions in dementia require a higher skill set from clinicians and researchers than that of simpler studies in order to deliver the right amount of information about how the intervention might work and the potential benefits at the most receptive time. Reported lack of insight into dementia diagnosis suggests that continually raising awareness of dementia, to reduce stigma and offering research opportunities as part of usual care as recommended, may increase dyads’ response to maximising independent living through research participation.^1^ Ultimately, the normalisation of trial participation could provide all service users with the opportunity to participate in research in the same way that they have a choice about their clinical care.

## Conclusion

There is no ‘one-size-fits-all’ approach to facilitating people with dementia and their carers’ research participation. Nonparticipation in research is an unavoidable reality; this study’s finding that people did not participate due to an imbalance of benefit in return for their level of involvement may not be representative of all potential dyads who do not participate in research. However, decliners can significantly affect the time and energy required to reach the appropriate audience and target sample during trial recruitment and possibly affect the generalisability of outcomes when published. Identifying and sharing the reasons why people declined participation can enable the tailoring of future psychosocial trials both at the design stage by enabling participants to choose their preferred arm and having a flexible tailored intervention in partnership with the dyad, and at the delivery stage by better targeting recruitment. These measures plus finely honed researcher skills to navigate the differing dyadic relationships, receptiveness to research opportunities, levels of acceptance of the dementia diagnosis and awareness of potential future challenges the dyad may face can potentially increase inclusivity and generalisability of evidence-based choices to meet the growing needs of society.

## Supplemental Material

VALID_trial_-_people_who_declined_to_participate_Indicative_Topic_Guide_v2_17.11.15 – Supplemental material for Reasons for nonparticipation in the Valuing Active Life in Dementia randomised controlled trial of a dyadic occupational therapy intervention: An interview study Click here for additional data file. Supplemental material, VALID_trial_-_people_who_declined_to_participate_Indicative_Topic_Guide_v2_17.11.15 for Reasons for nonparticipation in the Valuing Active Life in Dementia randomised controlled trial of a dyadic occupational therapy intervention: An interview study by Jacqueline Mundy, Jacki Stansfeld, Martin Orrell, Martin Cartwright and Jennifer Wenborn in SAGE Open Medicine
