# Supplementary material for: Reasons for nonparticipation in the Valuing Active Life in Dementia randomised controlled trial of a dyadic occupational therapy intervention: An interview study
Source: SAGE Open Med. 2020 Oct 15;8:2050312120958926. doi: 10.1177/2050312120958926 (PMC7576897; doi:10.1177/2050312120958926)
Supplement: VALID_trial_-_people_who_declined_to_participate_Indicative_Topic_Guide_v2_17.11.15 – Supplemental material for Reasons for nonparticipation in the Valuing Active Life in Dementia randomised controlled trial of a dyadic occupational therapy intervention: An interview study [file VALID_trial_-_people_who_declined_to_participate_Indicative_Topic_Guide_v2_17.11.15.pdf]

VALID Qualitative Interview Indicative Topic Guide – Family supporters not wanting to take part in VALID

- Introduce myself and confirm I am speaking with family supporter

"Thank you for the contact form you sent back, agreeing to take part in a short telephone call to further explore your reasons for deciding not to participate in the VALID study. Is now still convenient?"

"I will be recording our conversation today if that is OK, this is so I can really listen to all that you say and not miss anything"

- Await agreement and start recording

"Hopefully our conversation will identify ways to make research studies like VALID more acceptable to family carers and people with dementia, so I would like to thank you in advance for your time to help me today. During the conversation you can stop at any time if you've had enough and we can end it there or I can call back another time"

"To let you know, our conversation will be typed up and used both for my Masters Dissertation at City University and the VALID study team at NELFT. Your views will be anonymous; at no time will your personal details be attached to the interview notes or any materials we publish".

"So, thinking back to before you were approached for the VALID study…

1."What were your views about taking part in research before being invited to VALID?"

Prompts/further questions:

- Did you have any particular views beliefs relating to different types of research involvement?
- Where did they come from?

2. "What did you think taking part in the VALID study would involve for you both?"

Prompts/further questions:

- What interested you about VALID initially/ what were your first thoughts?
- Was VALID relevant to you? How/how not?
- Have you had community occupational therapy previously? Was it a positive/negative experience?
- What concerns/specific parts of VALID discouraged you from taking part? Why?

3."How did you come to the decision not to take part in VALID?"

Prompts/further questions:

- Did you make decision together/ alone or with others? Who helped?
- Did previous experience/s influence your decision/make it easier/harder to decide? In what way?
- Was the decision making process for taking part in the research any different to day-to-day decision making?

4 “The reason you gave for not taking part was [insert reason] please could you tell me more about this?”

Further questions related to the reason for declining to be added to relevant question as required.

Prompts/further questions:

- Is this the only reason why you decided not to take part?

Managing fine – to be used to expand on question 2, “What did you think participation in the VALID study would involve for you both?”

- Would there have been/ be a time when you might have wanted to take part?

I don’t want to take part in research - To be used to expand on question1, “What were your views about participating in research before being invited to VALID?”

- What types of research would you not want to take part in? Why?
- What type of research would you be interested in doing together/alone?

Busy – to be used to expand on question 3, “How did you come to the decision not to participate in VALID?”

- Who was it that was too busy? You, or the person with dementia that you support?
- What existing obligations and routines would VALID clash with?
- Were you too busy for research in general or the VALID intervention

Unwell - to be used to expand on question 3, “How did you come to the decision not to participate in VALID?”

- Who is it that is too unwell? You, or the person with dementia that you support?
- Would there be a time when you would have wanted to take part in VALID?

50:50 - To be used to expand on question 1, “What were your views about participating in research before being invited to VALID?”

- What were your concerns re the 50:50 random allocation? Why?

Other

- Questions to be related to the reason given.

I don’t want to give a reason – to be used to expand on question 3, “How did you come to the decision not to participate in VALID?”

- You advised you didn’t want to give a reason earlier, do you feel able to tell me now? then follow appropriate questioning stream

5. “What influence did the initial approach have on your decision not to take part?”

Prompts/further questions:

- How were you approached? Was [avenue of recruitment] timely/acceptable/ intrusive?
- Would a different approach have made a difference to you?
- Did you have too much/little information?

6. “What would make taking part in research easier for your particular circumstances?”

Prompts/further questions:

- What would have made you say yes?
- Would there have been a more acceptable type or research/type of intervention?

“That’s all my questions, do you have any questions for me?”

“Do you have anything else you would like to add?”

“Thank you very much for your time and contribution to the VALID study and my MSc. I shall now type up our conversation and review it to help inform the acceptability future dementia research that will aim to improve the provision of care.”
